# Profiling the expression of pro-metastatic genes in association with the clinicopathological features of primary breast cancer

**DOI:** 10.1186/s12935-020-01708-8

**Published:** 2021-01-06

**Authors:** Seyed-Mohammad Mazloomi, Mitra Foroutan-Ghaznavi, Vahid Montazeri, Gholamreza Tavoosidana, Ashraf Fakhrjou, Hojjatollah Nozad-Charoudeh, Saeed Pirouzpanah

**Affiliations:** 1 Nutrition Research Center, Department of Food Hygiene and Quality Control, Faculty of Nutrition and Food Sciences, Shiraz University of Medical Sciences, Shiraz, 7193635899 Iran; 2 Students’ Research Committee, Faculty of Nutrition and Food Sciences, Shiraz University of Medical Sciences, Shiraz, 7134814336 Iran; 3 Molecular Medicine Research Center, Biomedicine Institute, Tabriz University of Medical Sciences, Tabriz, 5166414766 Iran; 4 Department of Thoracic Surgery, Faculty of Medicine, Tabriz University of Medical Sciences, Surgery Ward, Nour-Nejat Hospital, Tabriz, 5166614766 Iran; 5 Department of Molecular Medicine, Faculty of Advanced Technologies in Medicine, Tehran University of Medical Sciences, Tehran, 1417755469 Iran; 6 Department of Pathology, Faculty of Medicine, Tabriz University of Medical Sciences, Tabriz, 5166614766 Iran; 7 Stem Cell Research Center, Tabriz University of Medical Sciences, Tabriz, 5166614756 Iran; 8 Department of Biochemistry and Dietetics, Faculty of Nutrition and Food Sciences, Tabriz University of Medical Sciences, Tabriz, 5166614711 Iran

**Keywords:** Breast cancer, Lymph node, Metastasis, Cortactin, RhoA, ROCK, Claudin

## Abstract

**Background:**

Metastasis accounts for ninety percent of breast cancer (BrCa) mortality. Cortactin, Ras homologous gene family member A (RhoA), and Rho-associated kinase (ROCK) raise cellular motility in favor of metastasis. Claudins (CLDN) belong to tight junction integrity and are dysregulated in BrCa. Thus far, epidemiologic evidence regarding the association of different pro-metastatic genes with pathological phenotypes of BrCa is largely inconsistent. This study aimed to determine the possible transcriptional models of pro-metastatic genes incorporate in holding the integrity of epithelial cell–cell junctions (*CTTN*, *RhoA*, *ROCK*, *CLDN-1*, *CLDN-2*, and *CLDN-4*), for the first time, in association with clinicopathological features of primary BrCa.

**Methods:**

In a consecutive case-series design, 206 newly diagnosed non-metastatic eligible BrCa patients with histopathological confirmation (30–65 years) were recruited in Tabriz, Iran (2015–2017). Real-time RT-PCR was used. Then fold changes in the expression of target genes were measured.

**Results:**

*ROCK* amplification was associated with the involvement of axillary lymph node metastasis (ALNM; OR_adj._ = 3.05, 95%CI 1.01–9.18). Consistently, inter-correlations of *CTTN*-*ROCK* (β = 0.226, P < 0.05) and *RhoA*-*ROCK* (β = 0.311, P < 0.01) were determined among patients diagnosed with ALNM^+^ BrCa. In addition, the overexpression of *CLDN*-*4* was frequently observed in tumors identified by ALNM^+^ or grade III (P < 0.05). The overexpression of *CTTN,* *CLDN*-*1,* and *CLDN*-*4* genes was correlated positively with the extent of tumor size. *CTTN* overexpression was associated with the increased chance of luminal-A positivity vs. non-luminal-A (OR_adj._ = 1.96, 95%CI 1.02–3.77). *ROCK* was also expressed in luminal-B BrCa tumors (P < 0.05). The estrogen receptor-dependent transcriptions were extended to the inter-correlations of *RhoA*-*ROCK* (β = 0.280, P < 0.01), *ROCK*-*CLDN*-*2* (β = 0.267, P < 0.05), and *CLDN*-*1*-*CLDN*-*4* (β = 0.451, P < 0.001).

**Conclusions:**

For the first time, our findings suggested that the inter-correlations of *CTTN*-*ROCK* and *RhoA*-*ROCK* were significant transcriptional profiles determined in association with ALNM involvement; therefore the overexpression of *ROCK* may serve as a potential molecular marker for lymphatic metastasis. The provided binary transcriptional profiles need more approvals in different clinical features of BrCa metastasis.

## Background

Breast cancer (BrCa) is globally the most common malignancy in women. BrCa is a heterogeneous disease with incident rate of 46.3 per 100,000 and mortality rate of 13.0 per 100,000 in 2018 worldwide, according to GLOBOCAN [1]. BrCa incidence in developing countries is increased due to cultural transition toward a sedentary lifestyle, Western diet, and increased rate of smoking, urbanization, and air pollutions [2–4]. It is accepted that estrogen leads to BrCa progression. Estrogen signaling is a therapeutic target for BrCa [5]; identifying pro-metastatic gene expression in different molecular subtypes (hormone receptors) with unique prognostic features may help elucidating new more personalized therapies. Furthermore, some important studies have documented evidence about preclinical diagnostic markers from predisposing mutations to raise the risk of BrCa [6], and the effect of some interventions on molecular targets have been assessed in solid tumors [7–10]. These could support lacking a consensus to support pro-metastatic genes in association with advanced features. Metastasis is considered for 90% of BrCa mortality which involves a complex multi-stage process, initially breaking away of tumor cells from primary tumor (dissociation step), degrading the proteins incorporate in the integrity of extracellular matrix (invasion step), transmigrate through vascular and/or lymph vessel (intravasation and extravasation steps), and cell-to-cell and cell–matrix adhesions with certain affinity to make organ-specific target metastasis (organotropism) [11].

Cortactin regulates actin cytoskeleton arrangement–a prerequisite for metastasis progressions—by binding to actin-related protein complex and facilitating releasing activated Wiskott Aldrich syndrome proteins [12]. Cortactin is an important regulator of cancer cell motility and mesenchymal movement [13]. Invadopodia, forming cellular actin-based protrusions, is mediated by cortactin activation and accompanies the invasion of cancer cells to the mesenchymal layer [14]. The overexpression of *CTTN* was associated with lymph node metastasis [15–19], advanced histologic grades [16, 20, 21], and larger tumor size [15, 16] in various cancers suggesting that cortactin might have prognostic impacts on different cancers but less paid attention to BrCa. One study reported a significant correlation between the protein expression of cortactin and lymphatic metastasis of breast tumors  [22]. Therefore, the *CTTN* expression in association with histologic grade and tumor size of BrCa is largely missing. Of the few earlier reports on cortactin expression in BrCa regarding molecular subtypes [23, 24], a meta-cohort of primary BrCa reported *CTTN* overexpression in hormone receptor-positive samples [24].

There is a bifunctional activity between cortactin and Ras homologous gene family member A/Rho-associated kinase (RhoA/ROCK) complex in integration for enhancing actin stress fiber formation [12, 25]. RhoA belongs to the small GTPase family [25]. It triggers cell motility and amoeboid movement via the extensive formation of actin stress fiber and actomyosin contractility regulation [25]. ROCK activity—a major downstream effector of RhoA—is to stabilize actin filament and phosphorylate myosin light chain to eventually raise reforming rates of actomyosin contractility [26]. *RhoA* or *ROCK* overexpression predicts shorter survival rates of BrCa [27, 28]. *RhoA* overexpression was observed in advanced histologic grade [29], larger tumor size [27], and stages II-III of BrCa [30]. *ROCK* overexpression was correlated with nodal involvement and advanced histologic grade of BrCa [28, 31]. Significant results were not reported regarding the association of *RhoA* and *ROCK* expression with lymphatic metastasis and tumor size of BrCa, respectively. Studies could rarely provide evidence to show the association of molecular subtype of BrCa and the expression of *RhoA* or *ROCK*.

Claudins (CLDNs)—membrane transport proteins—have critical functions in forming and maintaining cell-to-cell adhesion complexes, so-called tight junctions [32]. CLDNs generally connect to actin cytoskeleton, and their functions may be regulated by Rho/ROCK pathway [33]. CLDNs dysregulation is documented in epithelial-derived cancers [32]. Different isoforms of *CLDNs* present in a tissue-specific manner. Their various functions highly depend on cell’s microenvironment [34]. The protein expression of CLDN-1 was reported to be associated with poor prognosis [35], larger tumor size [36], and advanced histologic grade of BrCa [36, 37]. However, positive or negative protein expression of CLDN-1 was reported to be correlated to the lymph node metastasis of BrCa [36, 38]. While the positive protein expression of CLDN-2 was associated with poor prognosis [38, 39], its loss of expression was related to nodal involvement of BrCa [34]. No study reported a significant correlation between *CLDN-2* expression and histologic grade and tumor size of BrCa patients. Several reports indicated a positive protein expression of CLDN-4 connected to poor prognosis [37, 40], advanced grade [37, 40–43], and lymphatic metastasis [19, 41] of BrCa. However, positive or negative protein expression of CLDN-4 was reported to be correlated to a larger tumor size of BrCa [37, 43]. *CLDNs* expression was dependent on the molecular subtypes of breast tumors [35–38, 40, 41, 43, 44]. The data on the association of *CLDNs* expression and clinicopathological parameters remains inconclusive.

Sufficient information is not available to interpret pro-metastatic genes interaction in association with pathologic features in non-metastatic conditions. Therefore, this study aimed (i) to assess the correlation of pro-metastatic genes ‒ *CTTN, RhoA, ROCK, CLDN*-*1, CLDN*-*2,* and *CLDN*-*4* ‒ with clinicopathological parameters, (ii) to determine the inter-correlation of pro-metastatic genes in primary BrCa.

## Methods

### Study population

**Fig. 1:**
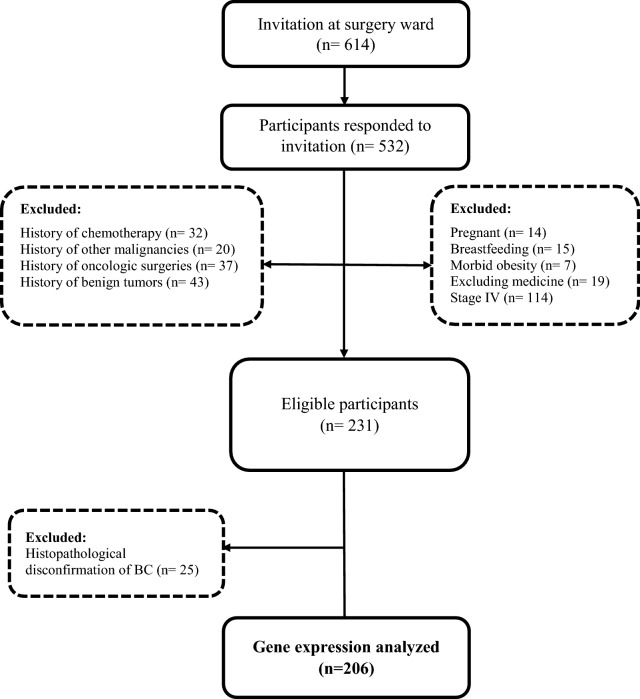
Flow chart diagram for the selection of study participants

This study is a part of ongoing large prospective cohort of consecutive case-series of BrCa patients-Breast Cancer Risk and Lifestyle (BCRL)- who were histopathologically diagnosed with primary malignancy. The BCRL is a multicenter study designed to assess lifestyle-related factors in association with BrCa risk prevention, regional to Northwestern Iran. The present study is a part of this cohort with ongoing recruitment began in May 2009. Participants with newly diagnosed and histologically confirmed BrCa (N = 206), who were admitted to the surgical wards of Nour-Nejat Hospital, Shams Hospital, Shahid Ghazi Educational-Oncology Hospital, and several oncology clinics located in Tabriz, Iran from May 2015 to January 2017. These are referral hospitals for oncologic surgeries with patients from different Northwestern Iran provinces (East and West Azerbaijan, Ardabil, Hamadan, and Zanjan). Participants were recruited from BrCa candidates before mastectomy surgery. The participants were 30-65 years old at the time of BrCa diagnosis. Eligible participants were mostly recruited from pre-menopausal women who had lymph node(s) positivity, invasive ductal carcinoma (IDC), and stages I-III. Other inclusion criteria mainly consisted of a completed written informed consent form and no subjective medical history, including benign breast diseases, other malignancy, and any oncologic surgery. Exclusion criteria were reported in our previous reports [3, 4, 45, 46] which were depicted in Fig. 1. Family history of breast and other cancers in first- and second-degree relatives were asked to lay out related pedigree analysis. Anthropometric measurements were examined as well.

### Pathologic data

**Fig. 2:**
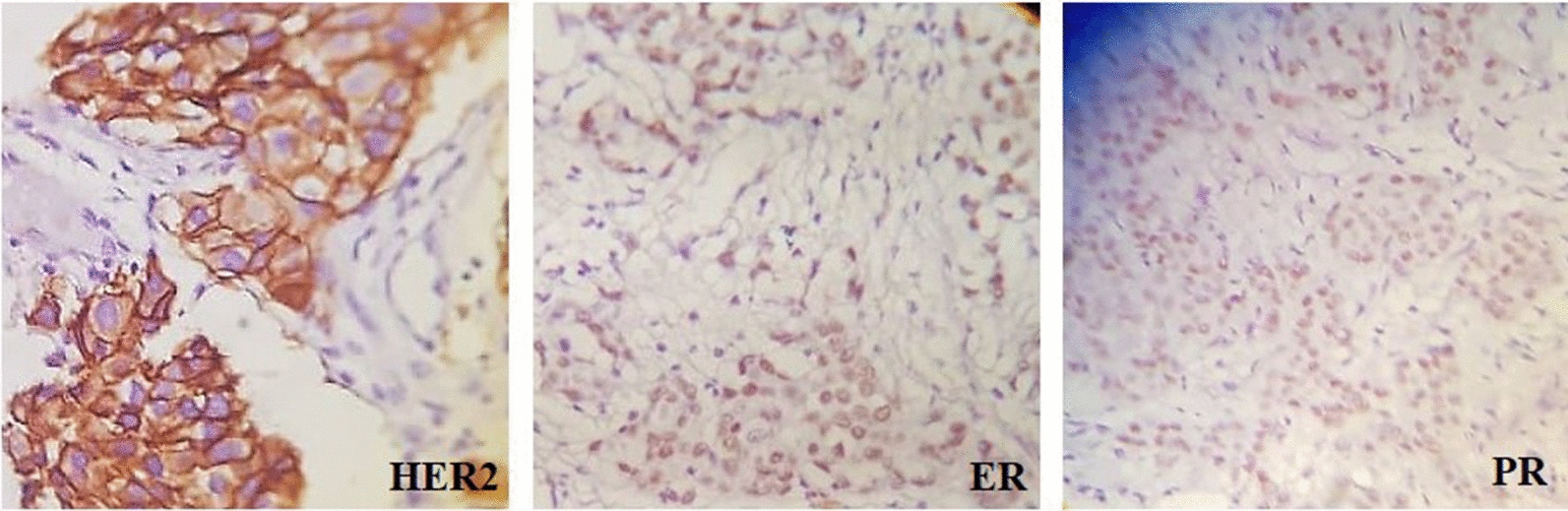
Immunohistochemical protein expression of human epidermal growth factor receptor 2 (HER2), estrogen receptor (ER), and progesterone receptor (PR). The original magnification was X400

Histopathologic data, including tumor size, histological subtype (IDC and non-ductal carcinoma), axillary lymph node metastasis (ALNM), and histological tumor grade, were obtained from objective medical records. The tumor size was considered the greatest diameter of tumor [47] Evaluating the histologic grades was determined by reviewing the stained microtones of paraffin-embedded tumor samples according to the Nottingham combined grading system to detect grades based on tubule formation, nuclear grade, and mitotic activity [47]. Immunohistochemical staining was carried out for human epidermal growth factor receptor-2 (HER2), estrogen receptor (ER), and progesterone receptor (PR) (Fig. 2). For HER2 positivity, the membrane and cytoplasmic staining ≥ 10% of breast tumor cells were considered weak or high intensity [47]. Immunohistochemistry results obtained by nuclear antibody staining when accounted for above 1% of tumor cells indicated the positivity of ER and PR staining [48]. Molecular subtypes were classified based on the protocol summarized by Wu et al. [47]. Clinical staging was defined according to the American Joint Committee on Cancer; 8th BC staging guidelines [49]. Neither BrCa patients with contralateral ALNM classified as distant metastases (M1) nor BrCa patients with distant metastasis (M1; stage IV) were recruited [49]. Some pathological data were not available due to missing.

### Quantitative real-time reverse transcriptase-polymerase chain reaction (qRT-PCR)

Before mastectomy surgery, fresh frozen tumor tissue and normal adjacent tissue were collected. Surgical tumor tissue section (core biopsy) consisted of 85% tumor cells with microscopic examinations [50]. QIAzol (Qiagen, USA) was applied to extract total mRNA and then evaluated using NanoDrop 2000 (Thermo Scientific, Germany), accounting for the absorbance at 260/280 nm. Total mRNA was converted into cDNA using QuantiTect reverse transcriptase (Qiagen, USA), based on the manufacturer’s protocol. The expression levels of target genes were determined by qRT-PCR, using LightCycler 480II (Roche, Germany). Materials used for PCR were composed of 12.5 μl from 10 × SYBER Green Master Mix (Nanohelix, South Korea), each primer solution (~ 10 pmol/μl), template cDNA (~ 100–200 ng/μl), and DEPC-treated water. PCR steps included an initial denaturation (15 min at 95 °C), followed by 40 cycles of denaturation (24 s at 95 °C) and annealing (35 s at 62 °C). Each sample was amplified in duplicate reactions. The amplification of *hypoxanthine*–*guanine phosphoribosyltransferase* was as an internal control (Additional file 1: Figure S1). Fold changes in the expression of target genes (*CTTN, RhoA, ROCK, CLDN*-*1, CLDN*-*2,* and *CLDN*-*4*) were calculated using a 2^−ΔΔct^ formula [51]. PCR primers for relevant genes were listed in Additional file 2: Table S1.

### Statistical analysis

A sample size including 158 subjects was calculated based on information provided by Dales et al. [52] regarding type I error (alpha) at 0.05 and the power of analysis (1 − β) at 90%. Outlier data were detected using the box plots. Kolmogorov–Smirnov test and histogram plot were carried out to assess the normal distribution of continuous variables. After using Chi square test, the correlation between two sets of categorical variables was interpreted. Fold change in the expression of gene was compared among sub-categories of clinicopathological (molecular subtypes, histologic grades, tumor size, and ALNM) by conducting the one-way analysis of covariance (ANCOVA) set at posthoc Bonferroni method, and the results were represented by bar diagram. Linear regression analysis was performed to present standardized β-coefficients (β) among genes in certain clinical stages (I and II-III) and binary status of hormone receptors, and ALNM features. Also, scatter plots were illustrated to show the correlations between targeted genes and tumor size. Standardized β out of linear regression analysis was accompanied by scatter plots in crude (β) and adjusted models (β_adj._). Fold changes in the expression of genes were dichotomized using (1) median values, and (2) cutoff points determined by plotting receiver operating characteristic (ROC) curve based on ALNM status (as reference). Odds ratio (OR) and 95% confidence interval (95% CI) were obtained by logistic regression analysis to explore interesting genes as independent determinants of clinicopathological outcomes. The primary criteria for selecting a confounder in a model were identifying a significant univariate analysis concerning dependent variable. A certain model was identified for each gene using multivariate logistic regression analysis. The adjusted confounding factors were listed in Additional file 3: Table S2. Statistical analyses were performed using SPSS software, version 16 (SPSS Inc., USA). All two-tailed P-values < 0.05 were considered significant.

## Results

The clinicopathological characteristics of 206 patients were summarized in Table 1. The mean age in diagnosing these patients was 46.65 ± 8.61 years. Patients were frequently younger than 24 years at first pregnancy (60.6%, P < 0.01) and were older than 13 years at menarche (80.1%, P < 0.001). They were pre-menopause (66.5%) and histopathologically identified with IDC (92.0%), ER + (87.3%), PR + (85.1%), and HER2- (79.6%) in the total study population (P < 0.001). Histologically, the most frequent dimension of the tumor was T2 (2 cm < size ≤ 5 cm) (61.3%, P < 0.001). Stage II BrCa was more frequent among the participants (65.7%). Histologic grade II tumors (67.4%) and ALNM involvement (65.2%) were the most observed histological features (P < 0.001). A significant agreement existed between BrCa frequency diagnosed with ALNM and lymphatic invasion status (P < 0.001), indicating the acceptable accuracy of data represented as ALNM status (Additional file 4: Table S3).

**Table 1:** Clinicopathological characteristics of patients with invasive BrCa (N = 206)

Variable	Total patients (n)	The relative frequency (%)	P-value*
Age at diagnosis (year)			
Mean ± S.D.	46.65 ± 8.61		
< 48	117	56.8	0.051
≥ 48	89	43.2	
Age at first pregnancy (year)			
Mean ± S.D.	22.11 ± 4.86		
< 24	120	60.6	*0.003*
≥ 24	78	39.4	
Age at menopause (year)			
Mean ± S.D.	47.86 ± 4.73		
Post-menopause	69	33.5	*<0.001*
Pre-menopause	137	66.5	
Age at menarche (year)			
Mean ± S.D.	13.52 ± 1.36		
< 13	40	19.9	*<0.001*
≥ 13	161	80.1	
Tumor type			
IDC	172	92.0	*<0.001*
Others	15	8.0	
Tumor size (cm)			
Mean ± S.D.	2.96 ± 1.35		
T1 (size ≤ 2)	59	31.7	*<0.001*
T2 (2 < size ≤ 5)	114	61.3	
T3 (size > 5)	13	7.0	
Histologic grade			
I	39	20.9	*<0.001*
II	126	67.4	
III	22	11.7	
Axillary lymph node metastasis			
Negative	64	34.8	*<0.001*
Positive	120	65.2	
Tumor stage			
I	55	30.4	*<0.001*
II	119	65.7	
III	7	3.9	
ER			
Negative	23	12.7	*<0.001*
Positive	158	87.3	
PR			
Negative	27	14.9	*<0.001*
Positive	154	85.1	
HER2			
Negative	144	79.6	*<0.001*
Positive	37	20.4	
Molecular subtype			
Luminal A (ER ± , PR ± , and HER2-)	135	74.6	*<0.001*
Luminal B (ER ± , PR ± , and HER2 +)	23	12.7	
HER2 rich (ER-, PR-, and HER2 +)	14	7.7	
Triple-negative (ER-, PR-, and HER2-)	9	5.0	

The statistically significant finding was shown in italics (*P *< 0.05)

*N *number, *S.D.* standard deviation, *ER* estrogen receptor, *PR* progesterone receptor, *HER2* human epidermal growth factor receptor 2, *IDC* invasive ductal carcinoma

*The *P-value* was obtained by the Chi square test.

Some missing existed in demographic and clinicopathologic data

**Fig. 3:**
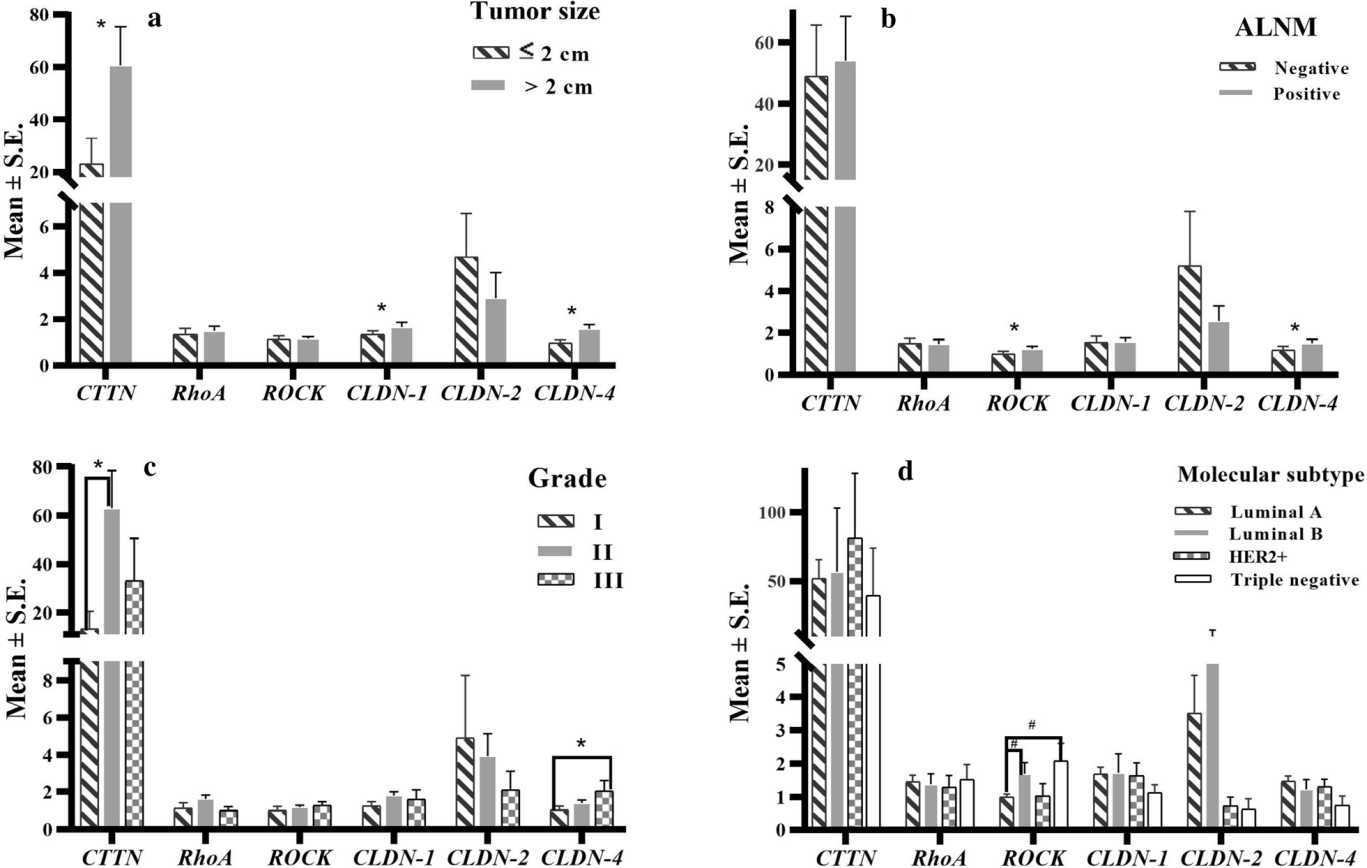
Comparing fold changes in the expression of studied genes (mean ± S.E.) across different histo-pathological features of non-metastatic primary BrCa patients. *P*-*values* were obtained using ANCOVA (post hoc Bonferroni method). *CTTN* cortactin, *RhoA* ras homolog gene family member A, *ROCK* rho-associated kinase, *CLDN* claudin, *S.E.* standard error, ALNM axillary lymph node metastasis, *HER2* human epidermal growth factor receptor 2. ^a^Adjusted for pregnancy _(number)_, lactation _(number)_, surgical treatment _(number)_, x-ray exposure _(number)_, BMI _(kg/m2)_, waist circumference _(cm)_, waist to hip ratio, and physical activity _(MET-h/week)_; ^b^Adjusted for age at menarche _(year)_, surgical treatment _(number)_, waist circumference _(cm)_, and physical activity _(MET-h/week)_; ^c^Adjusted for age at diagnosis _(year)_, duration of oral contraceptive usage _(month)_, lactation _(number)_, BMI _(kg/m2),_ hip circumference _(cm)_, and waist to hip ratio; ^d^Adjusted for the weight _(kg)_ and the age at menarche _(year)_. A significant result was indicated by *P < 0.05 or ^#^*P *< 0.01

Figure 3 illustrates bar diagrams comparing the fold changes in the expression of relevant genes among categorical dependent factors including tumor size (≤ 2, and > 2 cm), ALNM (+/-), histologic grades (I, II, and III), and molecular subtypes. Larger tumor size (> 2 cm) showed higher expression levels of *CTTN*, *CLDN*-*1*, and *CLDN*-*4* than those in smaller tumors (P < 0.05). Overexpressions of *ROCK* and *CLDN*-*4* were observed in ALNM + tumors more than BrCa counterparts lacking ALNM (P < 0.05). The expression levels of *CTTN* among the patients with grade II were higher than grade I (P < 0.05). There was an increasing trend in *CLDN*-*4* expression level among rising grades (P < 0.05). Of luminal A tumors, the fold change in the expression level of *ROCK* was found out less than those in luminal B and triple-negative tumors (P < 0.01).

**Fig. 4:**
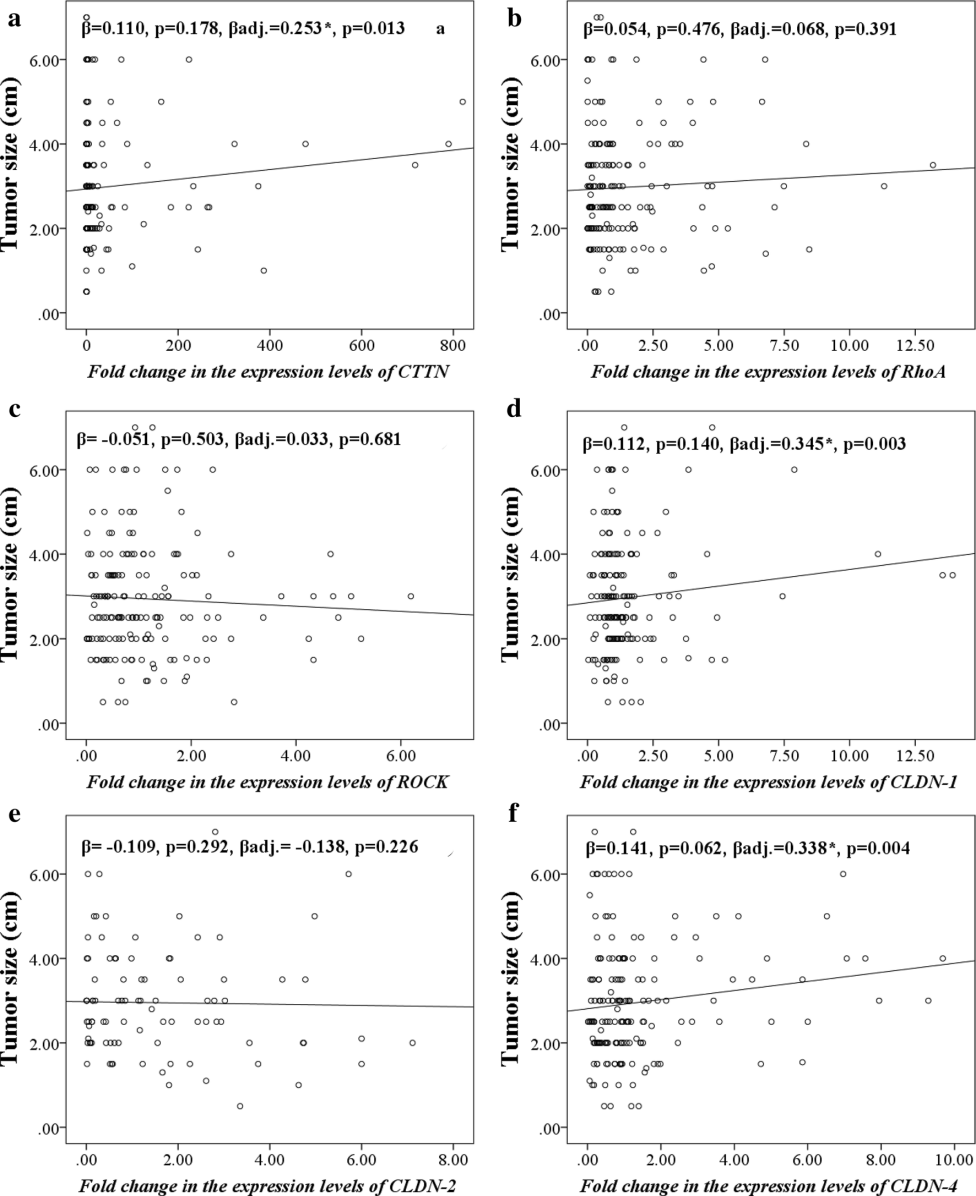
Scatter plots showing linear regression coefficients (standardized β) were depicted to present correlations between fold changes in the expression of studied genes and tumor size of BrCa  (n = 186). *CTTN* cortactin, *RhoA* ras homolog gene family member A, *ROCK* rho-associated kinase, *CLDN* claudin, βadj adjusted β. ^a^Adjusted for abortion _(number)_, pregnancy _(number)_, and hip circumference _(cm)_; ^b^Adjusted for age at diagnosis _(year)_ and BMI _(kg/m2)_; ^c^Adjusted for age at diagnosis _(year)_ and waist to hip ratio; ^d^Adjusted for abortion _(number)_, lactation _(number)_, the age at menarche _(year)_, and duration of oral contraceptive usage _(month);_^e^Adjusted for waist circumference _(cm)_ and age at first pregnancy _(year)_; ^f^Adjusted for age at menarche _(year)_, BMI _(kg/m2)_, abortion _(number)_, and duration of oral contraceptive usage _(month)_. A significant result was indicated by *P < 0.05

The scatter plots indicating the correlations among the genes of interest and tumor size were shown in Fig. 4. *CTTN* overexpression (β_adj._ = 0.253, P < 0.05), *CLDN*-*1* (β_adj._ = 0.345, P < 0.01), and *CLDN*-*4* (β_adj._ = 0.338, P < 0.01) were significantly correlated to the larger tumor dimension in the models adjusted for potential covariates.

OR and 95% CI estimated to represent the associations between the expression levels of genes and ALNM status (Table 2) and tumor grades (Table 3) using unadjusted (crude) and multivariate-adjusted models. According to high expression levels of *ROCK* in lymph node-positive (Fig. 3), *ROCK* overexpression was significantly associated with ALNM + after adjustments for potential covariates (OR _Median-based cutoff_ = 3.05, 95%CI 1.01–9.18) (Table 2). The overexpressions of *CTTN* (OR _ROC-based cutoff_ = 4.33, 95%CI 1.64–11.43) and *ROCK* (OR _ROC-based cutoff_ = 2.92, 95%CI 1.18-7.24) were associated with developed grade II breast carcinoma (Table 3). Multivariate adjusted models showed positive associations between *CTTN* (OR _ROC-based cutoff_ = 5.08, 95%CI 1.75–14.69), *ROCK* (OR _ROC-based cutoff_ = 2.86, 95%CI 1.14–7.14), and grade II tumors vs. considering grade I as reference (Table 3). Moreover, the overexpressions of *CTTN* and *ROCK* were associated with grade III in crude (*CTTN*: OR _ROC-based cutoff_ = 3.90, 95%CI 1.10–13.81; *ROCK*: OR _ROC-based cutoff_ = 4.40, 95%CI 1.33–14.48) and adjusted (*CTTN*: OR _ROC-based cutoff_ = 5.08, 95%CI 1.32–19.44; *ROCK*: OR _ROC-based cutoff_ = 4.22, 95%CI 1.26–14.07) models (Table 3).

**Table 2:** Odds ratios (ORs) and 95% confidence intervals (95%CI) presenting associations between fold changes in the expression of *CTTN*, *RhoA*, *ROCK*, *CLDN*-*1, CLDN*-*2,* and *CLDN*-*4* genes and axillary lymph node metastasis (ALNM) as a dependent pathological variable of primary non-metastatic BrCa-IDC (no distant metastasis) (n = 173)

Expression levels of gene	ALNM status							
					Crude OR (95%CI)		Adjusted OR (95%CI)	
	N	Negative	Positive	*P*-*value*^¥^	Negative	Positive	Negative	Positive
*CTTN*								
<2.31*	72	22 (30.6)^a^	50 (69.4)	0.282	1.00	0.77 (0.38–1.52)^b^	1.00	0.94 (0.46–1.92)^c^
≥2.31	77	28 (36.4)	49 (63.6)					
<4.88**	85	29 (34.1)	56 (65.9)	0.504	1.00	1.06 (0.53–2.11)	1.00	1.28 (0.62–2.64)^c^
≥4.88	64	21 (32.8)	43 (67.2)					
*RhoA*								
<0.69*	86	26 (30.2)	60 (69.8)	0.182	1.00	0.70 (0.37–1.33)	1.00	0.74 (0.39–1.40)^d^
≥0.69	87	33 (37.9)	54 (62.1)					
<0.73**	87	26 (29.9)	61 (70.1)	0.155	1.00	0.68 (0.36–1.28)	1.00	0.71 (0.37–1.35)^d^
≥0.73	86	33 (38.4)	53 (61.6)					
*ROCK*								
<0.83*	85	31 (36.5)	54 (63.5)	0.353	1.00	1.18 (0.63–2.23)	1.00	*3.05* *(1.01–9.18)*^*e*^
≥0.83	86	28 (32.6)	58 (67.4)					
<1.09**	104	39 (37.5)	65 (62.5)	0.195	1.00	1.41 (0.73–2.71)	1.00	2.18 (0.75–6.33)^e^
≥1.09	67	20 (29.9)	47 (70.1)					
*CLDN-1*								
<1.06 *	88	35 (39.8)	53 (60.2)	0.135	1.00	1.49 (0.79–2.81)	1.00	1.55 (0.82–2.95)^f^
≥1.06	85	26 (30.6)	59 (69.4)					
<0.75**	37	15 (40.5)	22 (59.5)	0.284	1.00	1.33 (0.63–2.81)	1.00	1.35 (0.63–2.87)^f^
≥0.75	136	46 (33.8)	90 (66.2)					
*CLDN-2*								
<1.15*	45	12 (26.7)	33 (73.3)	0.273	1.00	0.57 (0.23–1.37)	1.00	0.54 (0.21–1.37)^g^
≥1.15	49	19 (38.8)	30 (61.2)					
<1.12 **	15	6 (40.0)	9 (60.0)	0.363	1.00	1.44 (0.46–4.48)	1.00	1.60 (0.48–5.35)^g^
≥1.12	79	25 (31.6)	54 (68.4)					
*CLDN-4*								
<0.85 *	85	29 (34.1)	56 (65.9)	0.503	1.00	0.95 (0.50–1.78)	1.00	0.99 (0.52–1.87)^h^
≥0.85	88	31 (35.2)	57 (64.8)					
<0.67 **	67	28 (38.8)	41 (61.2)	0.229	1.00	1.34 (0.70–2.54)	1.00	1.37 (0.72–2.62)^h^
≥0.67	106	34 (32.1)	72 (67.9)					

The statistically significant finding is shown in *italic* fonts (*P *< 0.05)

*N* number, *OR* odds ratio, *CI* confidence intervals, *ALNM* axillary lymph node metastasis, *CTTN* cortactin, *RhoA* ras homolog gene family member A, *ROCK* rho-associated kinase, *CLDN* claudin

^a^Data were expressed as number (%)

^b^Logistic regression analysis was performed

Adjusted for ^c^Education status (illiterate/middle school/diploma/bachelor and higher) and waist circumference (< 90 cm/90 ≤ cm); ^d^Age at menarche (< 13 year/13 ≤ year) and waist circumference (< 90 cm/90 ≤ cm); ^e^Age at first pregnancy (< 24 year/24 ≤ year) and body mass index (≤ 24.99 kg/m^2^/25–29.99 kg/m^2^/30 ≤ kg/m^2^); ^f^Education status (illiterate/middle school/diploma/bachelor and higher) and body mass index (≤ 24.99 kg/m^2^/25–29.99 kg/m^2^/30 ≤ kg/m^2^); ^g^Education status (illiterate/middle school/diploma/bachelor and higher) and number of pregnancy (0-1/2-3/≥ 4); ^h^Age at menarche (< 13 year/13 ≤ year) and number of pregnancy (0-1/2-3/≥ 4)

^¥^Chi square test was performed

* Fold changes in the expression of studied genes were categorized based on median values

** Fold changes in the expression of studied genes were categorized based on the cutoff identified by Youden’s index

**Table 3:** Odds ratios (ORs) and 95% confidence intervals (95%CI) demonestrating associations between fold changes in the expression of *CTTN*, *RhoA*, *ROCK*, *CLDN*-*1, CLDN*-*2,* and *CLDN*-*4* genes and histologic grades as a dependent histopathological variable of primary non-metastatic BrCa (no distant metastasis) (n = 176)

Expression levels of gene	Grade												
						Crude OR (95%CI)				Adjusted OR (95%CI)			
	N	I	II	II	*P*-*value*^¥^	I	II	III	*P for trend*	I	II	III	*P for trend*
*CTTN*													
<2.31*	72	22 (30.6)^a^	42 (58.3)	8 (11.1)	*0.027*	1.00	*3.03* *(1.30–7.08)*^*b*^	3.02 (0.93*–*9.82)	*0.03*	1.00	*3.37* *(1.35–8.38)*^*c*^	*3.69* *(1.08–12.61)*	*0.020*
≥2.31	77	10 (12.7)	58 (73.4)	11 (13.9)									
<4.88**	86	26 (30.2)	50 (58.1)	10 (11.6)	*0.007*	1.00	*4.33* *(1.64–11.43)*	*3.90* *(1.10–13.81)*	*0.016*	1.00	*5.08* *(1.75–14.69)*^*c*^	*5.08* *(1.32–19.44)*	*0.010*
≥4.88	65	6 (9.2)	50 (76.9)	9 (13.8)									
*RhoA*													
<0.69*	86	22 (25.6)	55 (64.0)	9 (10.5)	0.238	1.00	1.77 (0.82*–*3.79)	2.27 (0.76*–*6.69)	0.107	1.00	1.81 (0.83*–*3.96)^d^	2.58 (0.86*–*7.76)	0.073
≥0.69	89	14 (15.7)	62 (69.7)	13 (14.6)									
<0.73**	87	22 (25.3)	55 (63.2)	10 (11.5)	0.305	1.00	1.77 (0.82*–*3.79)	1.88 (0.64*–*5.51)	0.184	1.00	1.81 (0.83*–*3.96)^d^	2.14 (0.72*–*6.38)	0.131
≥0.73	88	14 (15.9)	62 (70.5)	12 (13.6)									
*ROCK*													
<0.83*	85	21 (24.7)	55 (64.7)	9 (10.6)	0.347	1.00	1.66 (0.77*–*3.58)	2.00 (0.66*–*5.99)	0.172	1.00	1.65 (0.76*–*3.58)^e^	1.90 (0.63*–*5.74)	0.202
≥0.83	87	14 (16.1)	61 (70.1)	12 (13.8)									
<1.09**	105	28 (26.7)	67 (63.8)	10 (9.5)	*0.025*	1.00	*2.92* *(1.18–7.24)*	*4.40* *(1.33–14.48)*	*0.011*	1.00	*2.86* *(1.14–7.14)*^*e*^	*4.22* *(1.26–14.07)*	*0.014*
≥1.09	67	7 (10.4)	49 (73.1)	11 (16.4)									
*CLDN-1*													
<1.06 *	87	19 (21.8)	56 (64.4)	12 (13.8)	0.491	1.00	1.27 (0.60*–*2.69)	0.74 (0.24*–*2.25)	0.806	1.00	1.35 (0.63*–*2.89)^f^	0.78 (0.25*–*2.40)	0.891
≥1.06	89	17 (19.1)	64 (71.9)	8 (9.0)									
<0.75**	38	7 (18.4)	25 (65.8)	6 (15.8)	0.614	1.00	0.91 (0.36*–*2.33)	0.56 (0.15*–*1.99)	0.420	1.00	0.96 (0.37*–*2.48)^f^	0.57 (0.16*–*2.04)	0.452
≥0.75	138	29 (21.0)	95 (68.8)	14 (10.1)									
*CLDN-2*													
<1.15*	47	13 (27.7)	29 (61.7)	5 (10.6)	0.434	1.00	1.90 (0.69*–*5.23)	1.95 (0.44*–*8.54)	0.275	1.00	2.56 (0.79*–*8.34)^g^	2.99 (0.57*–*15.51)	0.143
≥1.15	48	8 (16.7)	34 (70.8)	6 (12.5)									
<1.12**	16	6 (37.5)	8 (50.0)	2 (12.5)	0.241	1.00	2.75 (0.82*–*9.15)	1.80 (0.29*–*10.90)	0.269	1.00	2.97 (0.84*–*10.44)^g^	2.04 (0.32*–*12.94)	0.243
≥1.12	79	15 (19.0)	55 (69.6)	9 (11.4)									
*CLDN-4*													
<0.85*	86	17 (19.8)	59 (68.6)	10 (11.6)	0.963	1.00	0.91 (0.43*–*1.92)	0.84 (0.33*–*2.89)	0.930	1.00	0.93 (0.43*–*2.01)^h^	1.04 (0.35*–*3.12)	0.980
≥0.85	90	19 (21.1)	60 (66.7)	11 (12.2)									
<0.67**	68	14 (20.6)	47 (69.1)	7 (10.3)	0.866	1.00	0.97 (0.45*–*2.09)	1.27 (0.41*–*3.93)	0.740		0.98 (0.45*–*2.14)^h^	1.32 (0.42*–*4.12)	0.687
≥0.67	108	22 (20.4)	72 (66.7)	14 (13.0)									

The statistically significant finding was shown in italics (*P *< 0.05)

*N* number, *OR* odds ratio,* CI* confidence interval, *CTTN* cortactin, *RhoA* ras homolog gene family member A, *ROCK* rho-associated kinase, *CLDN* claudin

^a^Data were expressed as number (%)

^b^Logistic regression analysis was performed

Adjusted for ^c^Education status (illiterate/middle school/diploma/bachelor and higher) and waist circumference (< 90 cm/90 ≤ cm); ^d^Age at menarche (< 13 year/13 ≤ year) and waist circumference (< 90 cm/90 ≤ cm); ^e^Age at first pregnancy (< 24 year/24 ≤ year) and body mass index (≤ 24.99 kg/m^2^/25-29.99 kg/m^2^/30 ≤ kg/m^2^); ^f^ Education status (illiterate/middle school/diploma/bachelor and higher) and body mass index (≤ 24.99 kg/m^2^/25-29.99 kg/m^2^/30 ≤ kg/m^2^); ^g^Education status (illiterate/middle school/diploma/bachelor and higher) and number of pregnancy (0-1/2-3/≥ 4); ^h^Age at menarche (< 13 year/13 ≤ year) and number of pregnancy (0-1/2-3/≥ 4).

^¥^Chi-square test was performed

*Fold changes in the expression of studied genes were categorized based on median values

**Fold changes in the expression of studied genes were categorized based on the cutoff identified by Youden’s index (ROC-based cutoff)

Since luminal A was the predominant sub-class of molecular subtypes in the present study, *CTTN* overexpression was significantly associated with luminal A vs. other molecular subtypes after adjustment for related confounders (OR _Median-based cutoff_ = 1.96, 95%CI 1.02–3.77) (Table 4). Tumors characterized by luminal B (vs. non-luminal B) was remarkable in tumors overexpressed *ROCK* (OR _ROC-based cutoff_ = 2.76, 95%CI 1.07–7.11). *ROCK* expression levels were also associated with triple-negative status compared to non-triple negative subtypes (OR _ROC-based cutoff_ = 6.29, 95% CI 1.27–31.11) (Table 4).

**Table 4:** Odds ratios (ORs) and 95% confidence intervals (95%CI) of fold changes in the expression of *CTTN*, *RhoA*, *ROCK*, *CLDN*-*1, CLDN*-*2,* and *CLDN*-*4* genes in association with molecular subtypes ^a^ of primary non-metastatic BrCa (no distant metastasis)  (N = 206)

Molecular subtype			Luminal B vs. Luminal A		HER2 rich vs. Luminal A		Triple-negative vs. Luminal A		Luminal A vs. Non-luminal A		Luminal B vs. Non-luminal B		HER2 rich vs. Non-HER2 rich		Triple-negative vs. Non-triple negative	
		N	23/129		13/129		9/129		129/78		23/183		13/194		9/198	
Gene	Status of expression		Crude OR (95%CI)	Adj. OR (95%CI)	Crude OR (95%CI)	Adj. OR (95%CI)	Crude OR (95%CI)	Adj. OR (95%CI)	Crude OR (95%CI)	Adj. OR (95%CI)	Crude OR (95%CI)	Adj. OR (95%CI)	Crude OR (95%CI)	Adj. OR (95%CI)	Crude OR (95%CI)	Adj. OR (95%CI)
*CTTN*																
	High/Low^*^	87/87	0.73 (0.26–2.03)^b^	0.61 (0.20–1.78)^c^	0.58 (0.17–1.96)	0.61 (0.18–2.05)^c^	0.32 (0.06–1.76)	0.36 (0.06–1.95)^c^	1.75 (0.93–3.28)	*1.96* *(1.02*–*3.77)*^h^	0.87 (0.32–2.39)	0.78 (0.28–2.21)^c^	0.69 (0.21–2.28)	0.74 (0.22–2.45)^c^	0.38 (0.07–2.04)	0.42 (0.08–2.27)^c^
	High/Low^**^	71/103	0.66 (0.22–1.91)	0.55 (0.18–1.69)^c^	0.86 (0.26–2.90)	0.92 (0.27–3.08)^c^	0.48 (0.09–2.61)	0.53 (0.10–2.90)^c^	1.68 (0.88–3.23)	1.79 (0.92–3.48)^h^	0.77 (0.27–2.19)	0.72 (0.25–2.09)^c^	1.03 (0.31–3.41)	1.08 (0.33–3.58)^c^	0.56 (0.10–3.01)	0.60 (0.11–3.21)^c^
*RhoA*																
	High/Low^*^	102/103	1.29 (0.52–3.22)	1.25 (0.50–3.15)^d^	1.73 (0.53–5.58)	1.73 (0.53–5.58)^d^	2.16 (0.51–9.03)	2.19 (0.52–9.16)^d^	0.83 (0.47–1.46)	0.82 (0.46–1.47)^d^	1.24 (0.51–3.01)	1.24 (0.50–3.02)^d^	1.66 (0.52–5.28)	1.67 (0.52–5.29)^d^	2.08 (0.50–8.56)	2.09 (0.50–8.62)^d^
	High/Low^**^	101/104	1.08 (0.43–2.67)	1.03 (0.41–2.60)^d^	1.73 (0.53–5.58)	1.73 (0.53–5.58)^d^	2.16 (0.51–9.03)	2.19 (0.52–9.19)^d^	0.87 (0.49–1.54)	0.87 (0.49–1.56)^d^	1.03 (0.42–2.50)	1.03 (0.42–2.50)^d^	1.70 (0.53–5.39)	1.70 (0.53–5.41)^d^	2.12 (0.51–8.74)	2.13 (0.51–8.81)^d^
*ROCK*																
	High/Low^*^	101/101	2.20 (0.82–5.90)	2.21 (0.81–5.99)^e^	1.02 (0.32–3.20)	1.19 (0.37–3.82)^e^	4.16 (0.83–20.82)	4.38 (0.86–22.25)^e^	0.62 (0.35–1.11)	0.59 (0.33–1.07)^i^	1.98 (0.75–5.20)	1.97 (0.74–5.22)^f^	0.84 (0.27–2.61)	0.78 (0.25–2.44)^f^	3.68 (0.74–18.19)	3.48 (0.70–17.26)^f^
	High/Low^**^	76/126	*2.83* *(1.07*–*7.43)*	*2.83* *(1.07*–*7.45)*^*e*^	1.17 (0.36–3.82)	1.25 (0.38–4.12)^e^	*6.60* *(1.31*–*33.14)*	*6.71* *(1.33*–*33.82)*^*e*^	0.71 (0.39–1.27)	0.66 (0.36–1.21)^i^	*2.76* *(1.07*–*7.11)*	*2.66* *(1.02*–*6.95)*^*f*^	1.03 (0.32–3.29)	0.91 (0.28–2.91)^f^	*6.29* *(1.27*–*31.11)*	*5.68* *(1.14*–*28.22)*^*f*^
*CLDN-1*																
	High/Low^*^	103/103	0.91 (0.37–2.22)	0.93 (0.38–2.27)^f^	1.16 (0.37–3.66)	1.20 (0.38–3.80)^f^	1.00 (0.24–4.17)	1.02 (0.24–4.30)^f^	1.00 (0.56–1.75)	0.99 (0.56–1.75)^g^	0.90 (0.38–2.16)	0.90 (0.37–2.16)^f^	1.17 (0.38–3.63)	1.18 (0.38–3.66)^f^	1.00 (0.24–4.11)	1.00 (0.24–4.12)^f^
	High/Low^**^	158/48	0.61 (0.22–1.63)	0.61 (0.22–1.64)^f^	1.47 (0.30–7.03)	1.48 (0.30–7.13)^f^	0.44 (0.10–1.98)	0.44 (0.10–2.00)^f^	1.37 (0.71–2.65)	1.39 (0.71–2.70)^g^	0.66 (0.25–1.71)	0.64 (0.24–1.68)^f^	1.72 (0.36–8.04)	1.68 (0.35–7.92)^f^	0.49 (0.11–2.13)	0.47 (0.10–2.08)^f^
*CLDN-2*																
	High/Low^*^	55/55	0.91 (0.27–3.12)	0.88 (0.26–3.03)^g^	0.36 (0.06–2.02)	0.34 (0.06–1.92)^g^	0.30 (0.03–3.08)	0.30 (0.03–3.05)^g^	1.27 (0.58–2.77)	1.30 (0.59–2.86)^g^	1.00 (0.30–3.31)	0.94 (0.28–3.14)^g^	0.37 (0.07–2.03)	0.36 (0.06–1.95)^g^	0.32 (0.03–3.18)	0.31 (0.03–3.17)^g^
	High/Low^**^	91/19	1.12 (0.21–5.73)	1.08 (0.21–5.62)^g^	1.34 (0.14–12.14)	1.28 (0.14–11.77)^g^	0.67 (0.06–6.99)	0.66 (0.06–6.92)^g^	0.81 (0.28–2.33)	0.81 (0.28–2.34)^g^	1.04 (0.21–5.22)^g^	0.89 (0.74–1.07)^g^	1.27 (0.14–11.20)	0.94 (0.77–1.13)^g^	0.61 (0.06–6.29)	0.61 (0.06–6.24)^g^
*CLDN-4*																
	High/Low^*^	103/103	0.69 (0.28–1.68)	0.72 (0.29–1.76)^g^	1.79 (0.51–6.25)	1.83 (0.52–6.40)^g^	0.53 (0.12–2.34)	0.54 (0.12–2.37)^g^	1.33 (0.75–2.35)	1.37 (0.77–2.42)^g^	0.74 (0.31–1.78)	0.77 (0.32–1.86)^g^	2.08 (0.60–7.15)	2.14 (0.62–7.36)^g^	0.58 (0.13–2.52)	0.35 (0.08–1.50)
	High/Low^**^	128/78	0.56 (0.23–1.38)	0.57 (0.23–1.42)^g^	2.58 (0.54–12.33)	2.65 (0.55–12.64)^g^	0.31 (0.07–1.36)	0.32 (0.07–1.41)^g^	1.52 (0.85–2.72)	1.54 (0.86–2.75)^g^	0.63 (0.26–1.50)	0.63 (0.26–1.52)^g^	3.22 (0.68–15.10)	3.24 (0.69–15.24)^g^	0.60 (0.14–2.61)^g^	0.35 (0.08–1.52)^g^

The statistically significant finding is shown in italics (*P *< 0.05)

*N* number, *OR* odds ratio, *CI* confidence interval, Adj adjusted, *CTTN * cortactin, *RhoA* ras homolog gene family member A, *ROCK* rho-associated kinase, *CLDN* claudin, *ER* estrogen receptor, *PR* progesterone, *HER2* human epidermal growth factor 2

^a^Molecular subtype: luminal A (ER ± , PR ± , and HER2-), luminal B (ER ± , PR ± , and HER2 +), HER2 rich (ER-, PR-, and HER2 +) and triple-negative (ER-, PR-, and HER2-) [45, 47]

^b^Logistic regression analysis was performed

Adjusted for ^c^Waist circumference (< 90 cm/90 ≤ cm); ^d^Body mass index (≤ 24.99 kg/m^2^/25-29.99 kg/m^2^/30 ≤ kg/m^2^); ^e^ Residence (city/rural); ^f^Age at first pregnancy (< 24 year/24 ≤ year); ^g^Number of pregnancy (0-1/2-3/≥ 4); ^h^ Waist circumference (< 90 cm/90 ≤ cm) and the age at first pregnancy (< 24 year/24 ≤ year); ^i^Age at menarche (< 13 year/13 ≤ year)

*Fold changes in the expression of studied genes were categorized based on median value

**Fold changes in the expression of studied genes were categorized based on the cutoff identified by Youden’s index (ROC-based cutoff)

Linear regression analysis was performed to obtain correlation coefficients (β) among the genes of interest and subgroup analyses due to hormonal receptor status, ALNM feature, and clinical staging, and presented in Table 5. For the total study population, findings indicated positive associations among the expression levels of *ROCK* and *RhoA* (β = 0.246, P < 0.001, *CTTN* (β = 0.170, P < 0.05), and *CLDN*-*2* (β = 0.237, P < 0.05) as dependent variables (Table 5). *CLDN*-*1* expression levels was strongly correlated to *CLDN*-*4* as well (β = 0.411, P < 0.001) (Table 5).

**Table 5:** Univariate linear regression analysis (standardized β coefficient) between the studied genes of tumors of study population of BrCa (N = 206)

	*CTTN* (n = 174)	*RhoA* (n = 205)	*ROCK* (n = 202)	*CLDN*-*1* (n = 206)	*CLDN*-*2* (n = 110)	*CLDN*-*4* (n = 206)
Total population						
*CTTN*	1					
*RhoA*	0.065 (0.395)*	1				
*ROCK*	*0.170 (0.028)*	*0.246 (< 0.001)*	1			
*CLDN*-*1*	− 0.036 (0.646)	− 0.007 (0.917)	− 0.062 (0.391)	1		
*CLDN*-*2*	0.043 (0.686)	− 0.017 (0.866)	*0.237 (0.014)*	0.034 (0.727)	1	
*CLDN*-*4*	0.013 (0.868)	0.086 (0.228)	− 0.023 (0.752)	*0.411 (< 0.001)*	− 0.102 (0.292)	1
ER- and PR ± (n = 23)						
*CTTN*	1					
*RhoA*	0.182 (0.465)	1				
*ROCK*	0.398 (0.091)	0.397 (0.067)	1			
*CLDN*-*1*	0.198 (0.415)	0.009 (0.967)	− 0.049 (0.832)	1		
*CLDN*-*2*	*0.655 (0.029)*	− 0.247 (0.465)	− 0.285 (0.396)	− 0.316 (0.344)	1	
*CLDN*-*4*	− 0.027 (0.914)	0.077 (0.748)	0.007 (0.975)	0.095 (0.691)	− 0.366 (0.299)	1
ER + and PR ± (n = 158)						
*CTTN*	1					
*RhoA*	0.074 (0.404)	1				
*ROCK*	0.128 (0.153)	*0.280 (0.001)*	1			
*CLDN*-*1*	-0.065 (0.469)	− 0.001 (0.994)	− 0.066 (0.433)	1		
*CLDN*-*2*	0.033 (0.786)	− 0.005 (0.965)	*0.267 (0.016)*	0.020 (0.858)	1	
*CLDN*-*4*	0.016 (0.859)	0.086 (0.301)	− 0.015 (0.862)	*0.451 (< 0.001)*	− 0.119 (0.284)	1
ALNM– (n = 64)						
*CTTN*	1					
*RhoA*	− 0.056 (0.705)	1				
*ROCK*	− 0.049 (0.741)	0.233 (0.083)	1			
*CLDN*-*1*	0.070 (0.638)	0.011 (0.932)	− 0.166 (0.217)	1		
*CLDN*-*2*	− 0.041 (0.847)	0.166 (0.382)	*0.733 (< 0.001)*	− 0.045 (0.810)	1	
*CLDN*-*4*	0.022 (0.882)	0.103 (0.446)	− 0.194 (0.151)	*0.522 (< 0.001)*	− 0.131 (0.490)	1
ALNM + (n = 120)						
*CTTN*	1					
*RhoA*	0.129 (0.204)	1				
*ROCK*	*0.226 (0.027)*	*0.311 (0.001)*	1			
*CLDN*-*1*	− 0.079 (0.448)	− 0.010 (0.921)	0.040 (0.681)	1		
*CLDN*-*2*	0.121 (0.384)	− 0.160 (0.219)	− 0.098 (0.443)	0.004 (0.976)	1	
*CLDN*-*4*	0.030 (0.769)	0.068 (0.479)	0.029 (0.763)	*0.377 (< 0.001)*	− 0.028 (0.826)	1
Stage I (n = 55)						
*CTTN*	1					
*RhoA*	0.060 (0.710)	1				
*ROCK*	*0.519 (0.001)*	0.252 (0.077)	1			
*CLDN*-*1*	− 0.025 (0.877)	− 0.060 (0.674)	− 0.115 (0.422)	1		
*CLDN*-*2*	− 0.064 (0.766)	0.204 (0.298)	*0.741 (< 0.001)*	− 0.049 (0.800)	1	
*CLDN*-*4*	0.232 (0.144)	0.039 (0.758)	0.002 (0.990)	*0.389 (0.004***)**	− 0.136 (0.480)	1
Stages II-III (n = 126)						
*CTTN*	1					
*RhoA*	0.098 (0.329)	1				
*ROCK*	0.069 (0.496)	*0.308 (0.001)*	1			
*CLDN*-*1*	0.002 (0.980)	0.022 (0.818)	0.002 (0.987)	1		
*CLDN*-*2*	0.130 (0.359)	− 0.163 (0.213)	− 0.130 (0.324)	0.013 (0.923)	1	
*CLDN*-*4*	− 0.052 (0.609)	0.085 (0.375)	− 0.019 (0.842)	*0.429 (< 0.001)*	− 0.014 (0.917)	1

The statistically significant finding is shown in italics (*P *< 0.05)

*N* number, *CTTN* cortactin, *RhoA* ras homolog gene family member A, *ROCK* rho-associated kinase, *CLDN* claudin, *ER* estrogen receptor, *PR* progesterone receptor, *ALNM* axillary lymph node metastasis

*Data were expressed as standardized β coefficient (*P*-*value*)

In the case of ER + tumors (PR +/-), *ROCK* up-regulation was significantly correlated to *RhoA* (β = 0.280, P < 0.01) and *CLDN*-*2* (β = 0.267, P < 0.05). Of tumors characterized by ER positivity, *CLDN*-*1* was significantly associated with *CLDN*-*4* (β = 0.411, P < 0.001) (Table 5). In ER-negative patients, the expression levels of *CTTN* and *CLDN*-*2* were strongly intercorrelated (β = 0.655, P < 0.05) (Table 5).

*ROCK* overexpression was significantly associated with up-regulation at *RhoA* (β = 0.311, P < 0.01) and *CTTN* (β = 0.226, P < 0.05) when there was the involvement of ALNM. The expression levels of *CLDN*-*1* and *CLDN*-*4* were inter-correlated in sub-population defined by the presence of ALNM  (β = 0.377, P < 0.001) and absent ALNM at diagnosis (β = 0.522, P < 0.001) (Table 5). In the absence of ALNM development, *ROCK* expression levels was strongly associated with *CLDN*-*2* (β = 0.733, P < 0.001) (Table 5).

Of patients at stage I, *ROCK* up-regulation was strongly correlated to *CTTN* (β = 0.519, P < 0.01) and *CLDN*-*2* (β = 0.741, P < 0.001) (Table 5). Moreover, *RhoA*-*ROCK* inter-correlation was significantly found out in clinical stages II-III (β = 0.308, P < 0.01). The overexpression of *CLDN*-*1* was significantly observed concerning *CLDN*-*4* among patients diagnosed with disease at stages I (β = 0.389, P < 0.01) and II-III (β = 0.429, P < 0.001) (Table 5).

## Discussion

For the first time, the results of this molecular epidemiologic study provided insights about the inter-correlations of *CTTN*-*ROCK* and *RhoA*-*ROCK* in association with the involvement of ALNM. It can substantiate *ROCK* over-regulation as a molecular determinant of tumor outgrowth and spread to axillary lymph nodes. The inter-correlation of *RhoA*-*ROCK* was associated with advanced clinical stages of primary BrCa. Moreover, the overexpression of *CTTN,* *CLDN*-*1,* and *CLDN*-*4* genes was positively correlated with the extent of tumor size, particularly in ER + status. *CLDN*-*4* up-regulation was notable in advanced histologic tumor grade and lymph node involvement.

### Lymphatic metastasis

A significant correlation was observed between *ROCK* overexpression and positive axillary lymph node involvement. Similar to our findings, Lane et al. [28] showed that protein and mRNA expression levels of *ROCK* were significantly correlated to nodal involvement. Bottino et al. [31] reported *ROCK* overexpression in breast tissue specimens (IDC) of patients who were diagnosed with ALNM. ROCK belongs to a family of serine/threonine kinases are recognized to promote actomyosin contractility by direct phosphorylation of myosin light chain [26]. Therefore, ROCK can promote the motility and adhesion of cancer cells in extravasation, thus might hold tumor dissemination possibility in lymphatic metastasis [53].

*RhoA* up-regulation was observed in tumors with lymph node metastasis in the cervical  [54] and colorectal cancers [55, 56]. An earlier study indicated *RhoA* overexpression in clinical stages II and III of BrCa [30]. The co-transcription of the *RhoA/ROCK* complex was reported in human cancers [54, 57]. Here, the co-transcription of *RhoA*-*ROCK* was observed in patients diagnosed with clinical stages II-III. This finding describes the possible contribution of RhoA and ROCK to potentiate tumor cells to develop invasive stages of BrCa, including local lymph node metastasis [53]. To the best of our knowledge, this is the first study that indicated *RhoA*-*ROCK* inter-correlation in association with ALNM +,  which supports the possible contribution of *RhoA* expression to its downstream effector molecule, i.e., the over-regulation of *ROCK,* thereby  likely to evoke the biological response related to metastasis [14, 25, 53].

Cortactin can potentially promote the polymerization and rearrangement of actin in the cellular cortex, which modulates actin cytoskeleton and related dynamics [13]. Findings of experimental animal models compared to MCF-7 tumor cells indicated that the mRNA level of *CTTN* might drive tumor cells to disseminate into lymphatic vessels and develop lymph node metastasis [58]. One study reported a significant correlation between the protein expression of cortactin and lymph node metastasis of breast tumors  [22]. The present findings provide new evidence showing that inter-correlation of *CTTN*-*ROCK* could be significantly correlated to positive lymph node involvement. In addition, cortactin effects on actin remodeling and subsequent degradation of the extracellular matrix occur in cancerous dissemination might be mediated by GTPase RhoA activity [59, 60]. Studies for supporting *CTTN*-*ROCK* interaction are limited; however, Croucher et al. [60] indicated that cortactin is competent to induce *RhoA* transcription in a dose-dependent manner in the case of *CTTN* overexpression which is revealed in head and neck squamous cells. A considerable dose of cortactin may overcome the distorted link between cortactin and RhoA by negative co-effectors [60]. Also, cortactin-related RhoA activity is documented to show cellular proliferation in head and neck squamous cell carcinoma [60]. GTP-RhoA (active form) interacts with the C-terminal part of coiled-coil domain and activates ROCK which is the main downstream target of active RhoA [26]. Nevertheless, a few pieces of evidence represented a background for cortactin-related RhoA activation, regardless of metastatic features, this study suggests evidence indicating the association between *CTTN* and *ROCK* expressions in favor of ALNM development.

The overexpression of *CLDN-4 *was observed in patients with lymphatic metastasis subgroup which is in agreement with previous reports of BrCa patients [19, 41]. The *CLDN*-*4*-dependent up-regulation of matrix metalloproteinase (MMP)-2 and MMP-9 and increased invasiveness might be another clue responsible for lymphatic metastasis [61].

### Histologic grades and tumor size

This is the first study to indicate the higher mRNA expression levels of *CTTN* in significant correlations with larger tumor size and histologic grade II of primary BrCa. Similarly, the mRNA expression levels of *CTTN* was correlated to a larger tumor size in colon cancer [15] and non-small cell lung cancers [16]. Moreover, previous studies reported that protein expression of cortactin could be associated with advanced histologic grades and poor differentiation in the cancers of colon [20], pancreas [21], and lung [16]. Overexpressed *CTTN* might induce the expression of S-phase kinase-associated protein-2 (SKP-2) to promote the cellular proliferation of head and neck squamous cell carcinoma [60], which was associated with larger tumor size observed in BrCa patients [62, 63]. Besides, cortactin-related SKP-2 signaling and subsequent down-regulation of cyclin-dependent kinase inhibitors might promote rapid cellular proliferation to result in increased tumor size. On the other hand, Clark et al. [64, 65] explained that cortactin is a transcriptional regulator of MMPs. Insulin-like growth factors and epidermal growth factor are potential tumor growing effectors released by MMP’s proteolytic activities, and likely MMPs can enhance cellular proliferation [66]. The findings indicated *CTTN* expression associated with larger tumor size of BrCa could describe cortactin as an effective variable and call to question its role in advanced histologic grade of breast carcinoma for future studies.

The present findings showed that mRNA expression of *CLDN*-*1* was positively correlated to larger tumor size in our population of BrCa. Similarly, in a previous study, the overexpression of *CLDN-1 *was reported in association with a larger tumor size [36]. CLDN-1 can interact with the epithelial to mesenchymal transition (EMT) related markers such as zinc finger protein SNAI-1 (Snail-1), zinc finger protein SNAI-2 (Slug), and zinc finger E-box binding homeobox-1 (Zeb-1) in human BrCa cell lines [44, 67, 68] and therefore can suppress E-cadherin which is an essential molecule incorporating into an active EMT. CLDN-1 might enhance Zeb-1 levels through phosphatidylinositol-3 kinase (PI3K)/protein kinase B (Akt) pathway and Wnt/β-catenin pathway to suppress E-cadherin-related EMT pathogenesis in colon cancer [69]. Furthermore, the overexpression of *CLDN*-*1* might be connected to MMP-9/Notch signaling to describe cellular proliferation in colorectal cancer [70]. Notch signaling is another aspect that gives rise to the overexpression of cyclins (A, B, and D) which they incorporate in cell cycle progression [71]. Moreover, overexpressed *CLDN*-*1* might improve MMP-2-mediated proliferation of vascular smooth muscle cells [72] which might enhance angiogenesis to integrate CLDN-1 to tumorigenesis. Also, the anti-apoptotic effect of CLDN-1 was indicated in tamoxifen-treated MCF-7 cell lines [73]. Our findings showed a positive correlation between *CLDN*-*4* expression and larger tumor size. Likewise, the positive protein expression of CLDN-4 was reported in correlation with larger tumor size of BrCa of Egyptian women [43]. CLDN-4 integration into tight junctions might be reduced by phosphorylation‒one of the post-translational modifications of CLDNs ‒ and therefore leads to gate function loss in various cancer cell lines [74, 75]. It might be a possible mechanism for *CLDN*-*4* overexpression in BrCa, as well. An in vivo assay in nude mice was transplanted by *CLDN*-*4*-silenced MCF-7 cells indicated the regression in breast tumor size [42]. A meta-analysis confirmed the elevated protein levels of CLDN-4 in larger tumor size in gastric carcinoma [76]. Consistent with earlier studies [37, 40–43], *CLDN*-*4* overexpression was associated with advanced histologic grade in present BrCa patients. The protein expression of CLDN-4 was positively correlated to Ki-67 labeling index among BrCa patients [37, 43], indicating that CLDN-4 might be contributed to proliferative pathways and cellular differentiation. Therefore, further studies in laboratory and epidemiologic outlooks are warranted to confirm further the association between CLDNs and advanced stages of BrCa.

### Hormone receptors

The higher mRNA expression level of *CTTN* was found in luminal A than non-luminal A subtypes. Accordingly, a large-scale cohort of primary BrCa patients indicated *CTTN* overexpression in association with BrCa metastasis in ER + samples [24]. Cortactin might increase the risk of breast adenocarcinoma metastasis to bone marrow mediated by hyaluronan/cluster of differentiation-44 (CD44) signaling in MCF-7 cell lines indicating that the expression level of *CTTN* can be positively regulated by CD44 [77]. Karamanou and colleagues [78] indicated less expression levels of CD44 and cortactin in ERα + MCF-7 than levels observed in ERβ + MDA-MB-231 BrCa cells [78]. Magalhaes et al. [79] reported that the tyrosine phosphorylation of cortactin increased recruitment of Na^+^/H^+^ exchanger-1 (NHE-1) in MDA-MB-231 cell lines. The overactivation of NHE-1 ‒ a plasma membrane glycoprotein that controls intracellular pH ‒ could result in an acidic extracellular microenvironment leading to breast tumor cell invasion and the development of metastasis [80]. It could collectively support our findings showing the overexpression of *CTTN* in luminal A subtype of BrCa, particularly when *CTTN* up-regulation was evident in pre-menopause BrCa patients.

The hyaluronan/CD44 signaling was also correlated to the activation of RhoA/ROCK pathway and subsequent the phosphorylation of NHE-1, leading to breast tumor cell invasion [81]. Consistent with Oviedo et al. [82] indicated that the presence of estradiol could result in increased protein and mRNA expression levels of *RhoA* in human umbilical vein endothelial cells; our findings represented an additional insight expressing the inter-correlation of *RhoA*-*ROCK* in association with ER positivity. We also demonstrated that the luminal B subtype was remarkable in tumors overexpressed *ROCK*, suggesting that high expression level of *ROCK* may be affected by HER2 expression of breast tumors. Exposure to physiological concentrations of 17β-estradiol in human umbilical vein endothelial cells resulted in the interaction between ERα and Gα13 (heterotrimeric G protein) to induce RhoA/ROCK activity [83]. The RhoA/ROCK signaling pathway could be activated by nuclear factor erythroid 2-related factor 2 (NRF-2) [84]. The treatment with estradiol could activate PI3K/glycogen synthase kinase-3 beta pathway to increase the activity of NRF-2 in MCF-7 cells [85]. A meta-analysis of clinical studies reported that *NRF2* overexpression was associated with a worse clinical outcomes of BrCa patients [86]. Another mechanism explains that in the presence of 17β-estradiol, ER may interact with c-Src to activate PI3K/Akt/RhoA pathway in human T47-D BrCa cells [87]. However, still, laboratory studies are insufficient to prove RhoA/ROCK bi-functionalities associated with the presence of certain molecular subtypes of BrCa.

ROCK may act as an upstream regulator to control *CLDNs* transcription [33]. The present study revealed a positive inter-correlation between *ROCK*-*CLDN*-*2* in the case of ER positivity of tumors. In addition, the inter-correlation of *CLDN*-*1*-*CLDN*-*4* was found abundant among the present ER + tumors. *CLDN*-*4* up-regulation was seen in the estrogen-related tissues such as the breast and ovaries [88, 89]. Blanchard et al. [44] reported protein expression of CLDN-1 in positive association with CLDN-4 in basal-like and non-basal breast tumors. Akimoto et al. [90] represented a positive correlation between the activity of estrogen-dependent G protein-coupled receptor 30/Akt-related pathway and *CLDN*-*1* expression in cervical adenocarcinoma cells [90]. The contradictory results from studies which addressed *CLDNs* expression concerning molecular subtypes [35–38, 40, 41, 43, 44], present findings could add an insight focusing on the association of *CLDN*-*1*-*CLDN*-*4* and *ROCK*-*CLDN*-*2* connected to breast tumors characterized by hormone receptor-positive.

The present significant correlation between *RhoA* and *ROCK* among breast tumors would give rise to the importance of *ROCK* transcription in accounting the present variation of *RhoA* expression. Several previous studies have considered *ROCK* expression as a reliable reference to determine the accuracy of *RhoA* expression in Her2-rich breast tissues [30], cervical cancer [54], and bladder cancer [57]. Consistent with the present findings, the protein expression of CLDN-1 was previously suggested as a biomarker to determine the accuracy of CLDN-4 expression in BrCa [44].

This study had some limitations. First, the sample size was small for subgroup analysis. Second, pathologic data were collected prospectively after surgery; therefore, data were not available for some cases. Third, the correction for multiplicity testing was a statistical approach warranted for future studies to provide better clusters of genes interdependently associated with the invasive pathological features of BrCa. Fourth, this study could not provide information about diagnostic accuracy based on the area under the curve of ROC, sensitivity, specificity, likelihood ratio, or other statistical parameters to interpret the diagnostic accuracy of pro-metastatic genes in detection or predicting BrCa features. Thereafter, we could not precisely address the accuracy of biomarkers to predict other disease diagnoses. The present findings provided some new evidence; further studies need to determine their prognostic impact on pathological characteristics in BrCa using a gold standard as reference. Accordingly, evidence should support pro-metastatic genes in association with advanced features before conducting any assessment for diagnostic accuracy of biomarkers. Microarray techniques could be suggested for future studies to assess a broader number of genes encompasses several pathways such as genes involved in controlling antioxidant defense system in association with *RhoA* transcription.

## Conclusions

In summary, findings could suggest the binary settings of pro-metastatic genes, including *CTTN*-*ROCK* and *RhoA*-*ROCK* in association with a breast tumor diagnosed with infiltration into axillary lymph nodes which is representative of local breast metastasis. Findings put emphasize on *ROCK *transcription as a contributor to ALNM of IDC -BrCa. The present findings indicated the overexpression of *CTTN, CLDN*-*1,* and *CLDN*-*4* in association with advanced stages of primary non-metastatic BrCa, which is highly evident in  ER + status of breast tumors. Endocrine therapy might correlate with ER/PR related pro-metastatic genes that need further implications by future studies. However, further experimental studies are necessary to reveal mechanisms underlying gene-to-gene interactions in association with the molecular events representative of metastatic hallmarks of BrCa.

## Supplementary information

**Additional file 1: Fig S1.** The overexpression of pro-metastatic genes were associated with clinicopathologic features of breast cancer. *CLDN* claudin, *CTTN* cortactin, *HGPRT* hypoxanthine–guanine phosphoribosyltransferase, *NTC* non-template control, *RhoA* ras homolog gene family member A, *ROCK* rho-associated kinase, N normal, *T* tumor.

**Additional file 2: Table S1.** Primer sequences used for qRT-PCR amplification.

**Additional file 3: Table S2.** General characteristics of patients with invasive breast cancer (N = 206).

**Additional file 4: Table S3.** Associations between lymphatic invasion and the involvement of axillary lymph node metastasis (ALNM).

## Data Availability

Data cannot be shared publicly due to legal restrictions imposed by the Ethics Committee of Tabriz University of Medical Sciences. Data are available by the Ethics Committee of Tabriz University of Medical Sciences (research-vice@tbzmed.ac.ir), for researchers who meet the criteria for access to confidential data.

## Abbreviations

ALNM: Axillary lymph node metastasis
ANCOVA: One-way analysis of covariance
BrCa: Breast cancer
BMI: Body mass index
CLDN: Claudin
CTTN: Cortactin
CI: Confidence interval
ER: Estrogen receptor
HER2: Human epidermal growth factor receptor-2
MMP: Matrix metalloproteinase
PR: Progesterone receptor
qRT-PCR: Quantitative real-time reverse transcriptase-polymerase chain reaction
OR: Odds ratio
RhoA: Ras homolog gene family member-A
ROCK: Rho-associated kinase
