# Supplementary figures and images for: Profiling the expression of pro-metastatic genes in association with the clinicopathological features of primary breast cancer
Source: Cancer Cell Int. 2021 Jan 6;21:6. doi: 10.1186/s12935-020-01708-8 (PMC7789694; doi:10.1186/s12935-020-01708-8)

**
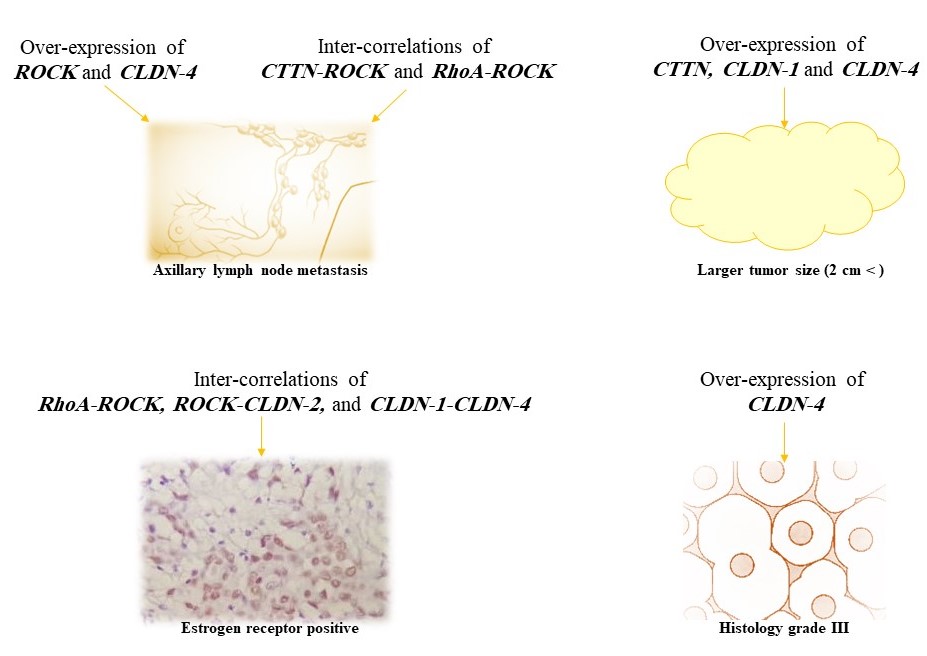
**
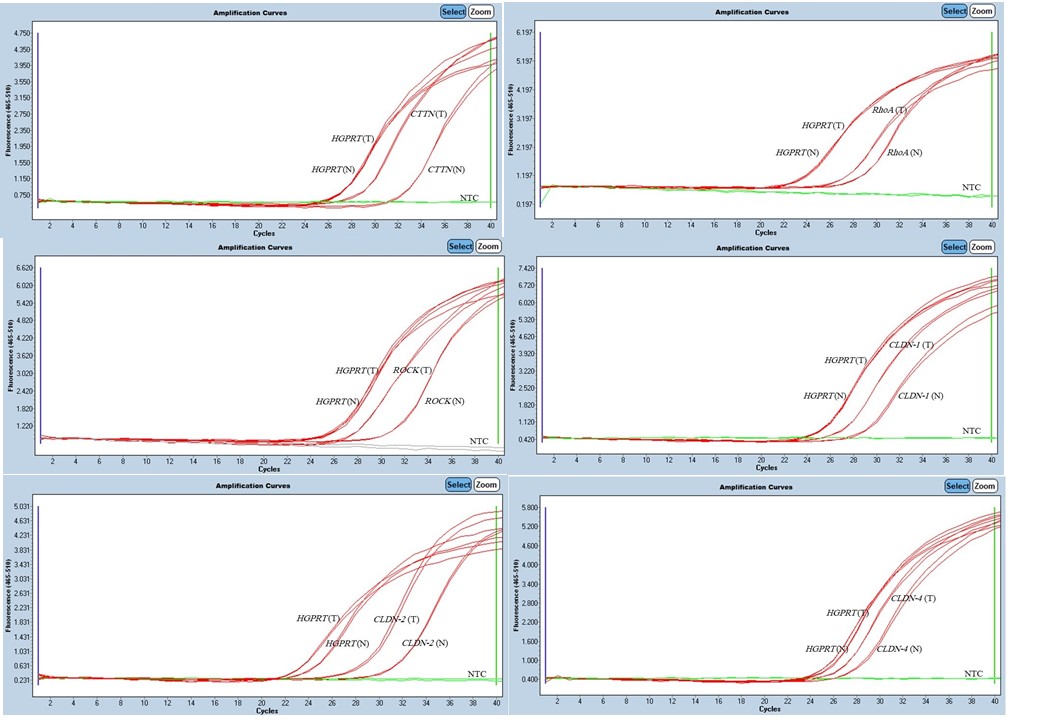

Supplement: Supplementary file 1 — Additional file 1: Fig S1. The overexpression of pro-metastatic genes were associated with clinicopathologic features of breast cancer. CLDN claudin, CTTN cortactin, HGPRT hypoxanthine–guanine phosphoribosyltransferase, NTC non-template control, RhoA ras homolog gene family member A, ROCK rho-associated kinase, N normal, T tumor. [file 12935_2020_1708_MOESM1_ESM.docx]
