# Supplementary material for: Profiling the expression of pro-metastatic genes in association with the clinicopathological features of primary breast cancer
Source: Cancer Cell Int. 2021 Jan 6;21:6. doi: 10.1186/s12935-020-01708-8 (PMC7789694; doi:10.1186/s12935-020-01708-8)
Supplement: Supplementary file 2 — Additional file 2: Table S1. Primer sequences used for qRT-PCR amplification. [file 12935_2020_1708_MOESM2_ESM.docx]

| Table S1. Primer sequences used for qRT-PCR amplification. | | | |
| --- | --- | --- | --- |
| Product size (bp) | Reverse primer | Forward primer | Genes |
| 111 | CCAGCAGCTCAGCAAAGAAT | TGGACAGGACTGAACGTCTT | *HGPRT* |
| 99 | CTGGTCACAGCTTCGACAGGTA | GCAGAAGGATCGGATGGTTAAG | *CTTN* |
| 84 | ATCGGTATCTGGGTAGGAGAGG | AAGCAGGTAGAGTTGGCTTTGTG | *RhoA* |
| 87 | AGCACGCAATTGCTCAATATCA | GCGCAATTGGTAGAAGAATGTG | *ROCK* |
| 102 | CTTCATACACTTCATGCCAA | CTGAGCAGCACATTGCAAGC | *CLDN-1* |
| 87 | CAGGCTGACTTCTCTCCTCC | CACAGGCCATTCAGGTCTTC | *CLDN-2* |
| 87 | GTGTGAGCAGACCAGTTCCT | TCTGCCAGAGCCATATAACT | *CLDN-4* |
| *HGPRT*, hypoxanthine-guanine phosphoribosyltransferase; *CTTN*, cortactin; *RhoA*, ras homolog gene family member A; *ROCK*, rho-associated kinase; *CLDN*, claudin; bp: base pair. | | | |
