# Supplementary material for: Profiling the expression of pro-metastatic genes in association with the clinicopathological features of primary breast cancer
Source: Cancer Cell Int. 2021 Jan 6;21:6. doi: 10.1186/s12935-020-01708-8 (PMC7789694; doi:10.1186/s12935-020-01708-8)
Supplement: Supplementary file 3 — Additional file 3: Table S2. General characteristics of patients with invasive breast cancer (N = 206). [file 12935_2020_1708_MOESM3_ESM.docx]

| **Table S2.** General characteristics of patients with invasive breast cancer (N=206) | | | |
| --- | --- | --- | --- |
| **Variables** | **Total patients (n)** | **The relative frequency (%)** | ***P- value**** |
| **Residence type** |  |  |  |
| City | 118 | 57.3 | **0.037** |
| Rural | 88 | 42.7 |  |
|  |  |  |  |
| **Education status**** |  |  |  |
| Illiterate | 28 | 13.6 | **<0.001** |
| Middle school | 81 | 39.5 |  |
| Diploma | 51 | 24.9 |  |
| Bachelor and higher levels | 45 | 22.0 |  |
|  |  |  |  |
| **Age at menarche (year)** ** |  |  | **<0.001** |
| < 13 | 40 | 19.9 |  |
| ≥ 13 | 161 | 80.1 |  |
|  |  |  |  |
| **Age at first pregnancy (year)** ** |  |  | **<0.001** |
| < 24 | 132 | 66.7 |  |
| ≥ 24 | 66 | 33.3 |  |
|  |  |  |  |
| **Pregnancy (number)** ** |  |  | **<0.001** |
| 0-1 | 33 | 16.4 |  |
| 2-3 | 118 | 58.7 |  |
| ≥ 4 | 50 | 24.9 |  |
|  |  |  |  |
| **Physical activity level** |  |  | **<0.001** |
| Sedentary | 25 | 12.1 |  |
| Low active | 55 | 26.7 |  |
| Active | 54 | 26.2 |  |
| Very active | 72 | 35.0 |  |
|  |  |  |  |
| **Body mass index (kg/m^2^)** ** |  |  | **<0.001** |
| ≤ 24.99 | 32 | 15.8 |  |
| 25-29.99 | 99 | 49.0 |  |
| 30 ≤ | 71 | 35.1 |  |
|  |  |  |  |
| **Waist circumference (cm)** ** |  |  | **<0.001** |
| < 90 | 48 | 24.2 |  |
| 90 ≤ | 150 | 75.8 |  |
| * The *P-value* was obtained by chi-square test.  ** Some missing existed in demographic and clinicopathological data.  Statistical significant finding is shown in bold (*P*<0.05). | | | |
