# Supplementary material for: Profiling the expression of pro-metastatic genes in association with the clinicopathological features of primary breast cancer
Source: Cancer Cell Int. 2021 Jan 6;21:6. doi: 10.1186/s12935-020-01708-8 (PMC7789694; doi:10.1186/s12935-020-01708-8)
Supplement: Supplementary file 4 — Additional file 4: Table S3. Associations between lymphatic invasion and the involvement of axillary lymph node metastasis (ALNM). [file 12935_2020_1708_MOESM4_ESM.docx]

| **Table S3.** Associations between lymphatic invasion and the involvement of axillary lymph node metastasis (ALNM). | | | |
| --- | --- | --- | --- |
| **Lymphatic invasion** | **ALNM** | |  |
|  | Negative | Positive | *P*-value |
| Negative | 38 (79.2) * | 13 (12.5) | <0.001 |
| Positive | 10 (20.8) | 91 (87.5) |  |
| The chi-square test was performed.  * Data expressed in the number of patients and the percentage in parenthesis. | | | |
